# Caregiving, ethnicity and gender in Māori and non‐Māori New Zealanders of advanced age: Findings from LiLACS NZ Kaiāwhina (Love and Support) study

**DOI:** 10.1111/ajag.12671

**Published:** 2019-05-16

**Authors:** Hilary Lapsley, Karen J. Hayman, Marama Leigh Muru‐Lanning, Simon A. Moyes, Sally Keeling, Richard Edlin, Ngaire Kerse

**Affiliations:** ^1^ Department of General Practice and Primary Health Care University of Auckland Auckland New Zealand; ^2^ James Henare Research Centre University of Auckland Auckland New Zealand; ^3^ Department of Medicine University of Otago Christchurch New Zealand; ^4^ Health Systems Section University of Auckland Auckland New Zealand

**Keywords:** aging, caregivers, ethnic groups, informal care, sex role

## Abstract

**Objective:**

This study investigates sex and ethnicity in relationships of care using data from Wave 4 of LiLACS NZ, a longitudinal study of Māori and non‐Māori New Zealanders of advanced age.

**Methods:**

Informal primary carers for LiLACS NZ participants were interviewed about aspects of caregiving. Data were analysed by gender and ethnic group of the LiLACS NZ participant.

**Results:**

Carers were mostly adult children or partners, and three‐quarters of them were women. Māori and men received more hours of care with a higher estimated dollar value of care. Māori men received the most personal care and household assistance. Carer employment, self‐rated health, quality of life and impact of caring did not significantly relate to the gender and ethnicity of care recipients.

**Conclusions:**

Gender and ethnicity are interwoven in caregiving and care receiving. Demographic differences and cultural expectations in both areas must be considered in policies for carer support.

**Policy Impact**

Female predominance in caregiving is a robust finding in ageing studies. That men, particularly Māori men, received more informal care suggests that more research is needed to tease out influential demographic and cultural factors, to underpin equitable carer support services.

## 1 INTRODUCTION

Caregiving in home settings is a cornerstone of “ageing in place” policies in New Zealand and many other countries.^1^, ^2^ Families and neighbours have long been the primary source of care for people in advanced age although, for social and demographic reasons, the pool of informal (usually unpaid) caregivers is diminishing even as need becomes greater.^2^ Ongoing shifts in the balance of care away from residential institutions are occurring and, while more home support services are now provided, informal caregiving remains the backbone of home care.

Research is fundamental to the development of policies providing supports to assist older people to live well at home even with frailty, disability or illness. Due to the gendered nature of caregiving,^3^ identifying patterns of sex and ethnicity in informal caregiving should be part of this research effort. The Kaiāwhina (Love and Support) study, a sub‐study of *Te Puāwaitanga O Ngā Tapuwae Kia Ora Tonu/ Life and Living in Advanced Age, a Cohort Study in New Zealand* (LiLACS NZ), provides research data that are relevant to understanding how gender and ethnicity relate to informal caregiving.^4^, ^5^, ^6^

The title “Kaiāwhina” is a Māori term that translates into English as “help” or “helper” but has complex overtones, drawing on notions of reciprocal relationships and obligations to help.^7^ The Kaiāwhina sub‐study provides data on primary carers nominated by LiLACS NZ participants and enables estimates of the economic value of care provided to be calculated.^5^

This study has a unique contribution to make to international research on caregiving. LiLACS NZ is the most sizeable New Zealand study of people in advanced age and, with its cohort of Māori New Zealanders, is the first longitudinal study worldwide of an indigenous population in advanced age.^4^ Its Kaiāwhina sub‐study has access to comprehensive data on those cared for via matching of participants. For the purposes of this paper, LiLACS NZ data allow fresh investigation of sex and ethnicity amongst informal carers and care recipients.

The research literature emphasises the psychosocial nature of informal caregiving, “characterised by relationships and social expectations” (p.4).^3^ Sex differences in informal caregiving have been identified, but not adequately investigated, especially in relation to the social expectations that place a disproportionate load of caring on female spouses and daughters of ageing parents.^8^, ^9^ Ethnicity differences in informal caregiving and its impacts are less explored. There are well‐recognised differences around family relationships, duties and obligations, ageing, illness and death in different cultures. Research in this field must be based on culturally relevant theorising and sound data however, rather than extrapolating from assumptions about attitudes of different cultures to older people and their role in families.^10^, ^11^, ^12^

While there is some research literature on caregiving in New Zealand, particularly in relation to caregiver stress,^13^ there is little focusing on sex, ethnicity and their interactions in caregiving, especially for those of advanced age. The New Zealand Longitudinal Study of Ageing found that women and Māori provided higher levels of care.^14^ That women provide the most care arises from gendered role expectations and, in later life, from their longevity and the fact that their spouses or partners tend to be older.^5^

In terms of ethnicity, there is scant literature specifically focusing on contemporary Māori caregiving, as compared with the broader literature around Māori families (whānau) and relationships. This may be partly because the duty of care is holistically embedded in the values of whānau, emphasising obligations and reciprocal relationships within the whānau and wider groupings.^7^, ^15^, ^16^ Caring amongst Māori may involve some different emphases to non‐Māori caring. One of the few extant studies showed that Māori carers facilitated spiritual guidance, acted as interpreters and advocates and maintained community links for those cared for, as well as undertaking the usual tasks.^16^ Recently published research associated with LiLACS NZ, focusing on end of life care, identified difficulties for Māori carers in navigating support.^17^, ^18^ A recent international study noted that an overarching theme in studies of indigenous end‐of‐life care was preparation of the spirit for the afterlife.^19^

### 1.1 Objective

The aim of this research was to investigate the influence of gender and ethnicity on informal care, via patterns of sex differences and ethnicity in Wave 4 data from informal carers in the LiLACS NZ Kaiāwhina study of carers for New Zealand Māori and non‐Māori women and men in advanced age. Carer data were matched to sex and ethnic status of LiLACS NZ participants in order to identify patterns of carer characteristics, their connection to LiLACS NZ participants, employment and benefit status, amount and type of care provided, feelings of responsibility and the impact of caring on employment, health and quality of life as well as overall positive and negative impact of care.

## 2 METHODS

The LiLACS NZ is a longitudinal study of two cohorts of Māori and non‐Māori women and men in advanced age. The study used a population‐based recruitment strategy identifying all non‐Māori aged 85 years and all Māori aged 80‐90 years in 2010 from one region of New Zealand. The age band for Māori enabled enrolment of sufficient numbers for meaningful analysis. Recruitment was informed by the New Zealand Electoral Roll to fulfil age and ethnicity criteria, with additional names obtained through word of mouth, advertising, residential care networks and personal contacts.^20^

Putting together the Māori cohort of LiLACS NZ was a dedicated process, involving extensive, local Māori‐led consultation to engage with Māori participants, as well as the creation of a governance group to protect principles of conduct for Māori in research.^20^ The non‐Māori cohort was mainly of Pakeha ethnicity (New Zealanders of European extraction).

Comprehensive health and social data were gathered from the LiLACS NZ participants by trained interviewers using standardised techniques.^4^ Enrolled participants were interviewed yearly, Wave 1 at baseline, Wave 2 after 12 months and so forth.

### 2.1 Selection of carers

The LiLACS NZ participants at Waves 3 and 4 were asked to identify the person who had provided them with the “most help, care and support in the last three months” and agree to that person being approached by the researchers. Carers could be a family member (including their spouse/partner), friend, neighbour or other unpaid helper, or a formal (employed) carer. If a formal carer was nominated, LiLACS NZ participants were asked if anyone else gave them care and if the second nomination was an informal carer, that person was approached for the Kaiāwhina sub‐study. Carer data used in this report come from informal carers only.

Wave 4 data were particularly comprehensive and give rise to the data used in this report. Altogether, 319 out of 438 Wave 4 participants (73%) consented to a carer being approached, with no significant difference between Māori and non‐Māori cohorts, although men (77%) were more likely to agree to carer contact than were women (71%). Some participants (8%) received no care, and they had significantly higher physical health‐related quality of life than the rest.^5^

Of the 286 carers who completed interviews, 261 (91%) were informal carers whose LiLACS NZ match equated to around 60 per cent of the 438 participants remaining at Wave 4 of the study. Of the matched LiLACS NZ participants (henceforth care recipients), women (73%) and non‐Māori (74%) predominated.

### 2.2 Interview topics and measures

The Kaiāwhina questionnaire for informal carers asked about age, ethnic group, sex, occupational and beneficiary status. Kaiāwhina (henceforth carers) were also asked about their relationship and residential proximity to the care recipient, about the types of care they gave, hours spent, how long they had been giving care and how much responsibility they felt in relation to the care they gave. Occupational and benefit status were recorded, and carers were asked whether caregiving impacted on their paid work. Government‐funded benefits are available for eligible New Zealanders and include a Family Funded Care benefit, paid to family members caring for someone with high health or aged care needs, as well as benefits unrelated to the care role such as disability support. Only two per cent of our carers reported receiving a benefit related to caring. Carers were also asked to rate their own health and to complete the EQ‐5D‐3L, a health status questionnaire designed to measure health‐related quality of life.^21^ The study also used the Carers of Older People in Europe (COPE) index, a measure of carer support and carer stress with subscales measuring positive values and negative impacts of caregiving.^22^

### 2.3 Analysis of data

Descriptive statistics are used to describe the carers and their matched care recipients. The matching made it possible to analyse carer data in relation to care recipient characteristics by sex and ethnicity. Differences between groups were tested using Fisher's exact test, chi‐square and t tests. In relation to COPE scores, Probit regression was used to examine carer scores as the dependent variable against sex and ethnic group of care recipients. Responses for weekly caring time were recorded categorically (as <3, 4‐9, 10‐19, 20‐49 and 50+ hours); midpoints of each category were used in the analysis except for the open ended category (60 hours used).^5^

## 3 RESULTS

### 3.1 Carers and matched LiLACS NZ participants

Table 1 presents data on carer age, ethnic group and sex in relation to female and male Māori and non‐Māori care recipients. While non‐Māori care recipients in Wave 4 were in their late 80s, Māori care recipients had a wider age range (mid‐80s to mid‐90s) as per the recruitment criteria for LiLACS NZ. The mean age of carers was 66 years, with carers for Māori on average three years younger than carers for non‐Māori (64 years compared to 67 years). Around three‐quarters of carers were women.

**Table 1:** Informal carer characteristics by matched care recipients

	LiLACS NZ: Māori		LiLACS NZ: Non‐Māori		LiLACS NZ: All	*P*‐value for sex	*P*‐value for ethnicity
	Women	Men	Women	Men			
Age (years) of care recipients, mean (SD)	85.5 (2.7)	85.2 (2.6)	87.6 (0.5)	87.7 (0.5)	86.8 (2.0)	1.0	<0.001
Characteristics of carers; n = 261							
Age (years), mean (SD)	63.5 (12.0)	66.0 (12.8)	66.5 (12.3)	68.8 (13.3)	66.3 (13.1)	0.1	0.08
Ethnic group, n (%)							
Māori, n (%)	40 (73)	23 (66)	2 (2)	1 (1)	66 (26)	0.4	<0.001
Non‐Māori, n (%)	15 (27)	12 (34)	83 (98)	78 (99)	188 (74)		
Sex: female, n (%)	36 (67)	33 (89)	56 (63)	65 (81)	190 (73)	<0.001	0.4

Abbreviations: LiLACS NZ, Life and Living in Advanced Age, a Cohort Study in New Zealand; SD, standard deviation.

In relation to caring across sex and ethnicity divides, more than four out of five male care recipients, compared to around two‐thirds of female care recipients, had a female carer. Thirty per cent of the LiLACS NZ Māori care recipients had carers who were non‐Māori, whereas few non‐Māori care recipients had Māori carers (2%).

Table 2 presents data on the relationship of carers to care recipients, distance lived from care recipients and employment and benefit status. Eighty‐nine per cent were family members, including in‐laws. Twenty‐six per cent were spouses/partners (average age 81), 56 per cent children (average age 58), and 7 per cent other family members (average age 58). The average age of non‐family carers (11% of the total) was 75 years. Around two‐thirds of carers for the women care recipients were their children, as compared with around half for the men (similarly for both Māori and non‐Māori).

**Table 2:** Informal carers: Relationships, distance, employment status and benefit receipts by ethnic group and sex of care recipients

	LiLACS NZ Māori, n (%)		LiLACS NZ: Non‐Māori, n (%)		LiLACS NZ: All n (%)	*P*‐value for sex	*P*‐value for ethnicity
	Women	Men	Women	Men			
Relationship to care recipient							
Spouse	9 (17)	17 (47)	12 (14)	37 (47)	97 (26)	<0.001	0.8
Children	34 (63)	16 (44)	59 (68)	36 (46)	205 (56)		
Other family	5 (9)	2 (6)	3 (3)	4 (5)	26 (7)		
Other	6 (11)	1 (3)	13 (15)	2 (3)	40 (11)		
Distance from care recipient							
Live in the same house/or house on same property	28 (51)	29 (78)	23 (26)	42 (53)	122 (47)	<0.001	<0.001
Live 30 min away or less	24 (44)	6 (16)	53 (60)	31 (39)	114 (44)		
Live more than 30 min away	3 (6)	2 (5)	12 (14)	7 (9)	24 (9)		
Employment status							
Full time	17 (31)	9 (24)	23 (26)	29 (36)	78 (30)	0.6	0.2
Part time	8 (15)	3 (8)	24 (28)	8 (10)	43 (17)		
Not employed (includes retired, fulltime student)	30 (55)	25 (68)	40 (46)	43 (54)	138 (53)		
Receipt of benefit							
Carer's benefit	3 (6)	2 (5)	0 (0)	0 (0)	5 (2)	0.8	<0.001
Other benefit	3 (6)	5 (14)	1 (1)	2 (3)	11 (4)		
No benefit or other support	49 (89)	30 (81)	87 (99)	78 (98)	244 (94)		

Most carers lived close to the person they cared for (47% in the same house or on the property, 44% within a 30‐minute drive, and only 9% more than 30 minutes away). Those caring for Māori were significantly more likely to live in the same house or on the property (there were more extended family situations), as were carers for men (men had more carers who were spouses/partners).

Around half of carers were employed full or part time, more so carers for non‐Māori than Māori, although this difference was not significant. Virtually no carers received a carer's benefit.

### 3.2 Amount and type of care

Table 3 shows that caring began, on average, 12 years before the interviews took place. LiLACS NZ men, both Māori and non‐Māori, had been cared for significantly longer than LiLACS NZ women (15 years on average, compared to 10 years).

**Table 3:** Amount and type of care from informal carers by ethnic group and sex of care recipients

	Māori		Non‐Māori		*P*‐value for sex	*P*‐value for ethnicity
	Women	Men	Women	Men		
Number responding	54	37	88	78		
Time in years since initiation of care, all informal carers, mean (SD)	10.28 (14.84)	15.59 (20.90)	9.17 (10.68)	14.14 (20.01)	0.02	0.6
Hours of care per week, all informal carers, mean (SD)	21.68 (20.76)	29.08 (19.33)	12.30 (15.03)	18.85 (19.75)	0.003	<0.001
Hours of care, spouse	27.56 (16.58)	32.62 (19.85)	37.33 (19.28)	34.37 (18.48)	1.0	0.358
Hours of care, child	22.64 (22.29)	24.13 (18.29)	8.53 (9.86)	4.81 (6.17)	0.4	<0.001
Hours of care, other family	10.10 (13.87)	47.25 (18.03)	5.83 (7.51)	6.00 (6.14)	0.07	0.04
Hours of care, other	10.83 (12.53)	6.50(–)	8.19 (9.53)	18.00 (23.33)	0.5	0.9
Carer feels all responsibility for participant, n (%)	14 (25)	14 (37)	13 (15)	14 (17)	0.2	0.008
Types of care given daily or several times a week						
Personal, n (%)	13 (24)	13 (35)	10 (11)	14 (17)	0.09	0.009
Household assistance, n (%)	34 (66)	29 (77)	34 (38)	44 (54)	0.007	<0.001

Men received significantly more hours of care than women on a weekly basis. Māori men received on average the most hours per week of care (29 hours compared to 19 hours for non‐Māori men, 22 hours for Māori women and 12 for non‐Māori women). Spouses/partners gave the most care, averaging over 30 hours per week, the male spouses of non‐Māori women estimating the highest number of hours of care at 37 hours. Offspring in the primary carer role gave around three times as many hours of care for Māori than for non‐Māori parents.

Nearly a fifth of the carers said that they had all the responsibility for caregiving. Spouses were significantly more likely than children to feel that they had all the responsibility (27% compared to 15%). A significantly higher proportion of those caring for Māori men (37%) felt that they had all the responsibility.

Carers were asked about types of care provided to care recipients over the last three months. Virtually all carers provided social and emotional support. Approximately three‐quarters provided at least some household assistance, such as help with shopping, transport, laundry, preparing meals, household chores, gardening and home maintenance, more than half of these every day or several times a week. One‐third (33%) provided at least some personal care, such as help with dressing, washing, eating, taking medication or toileting, around one in five daily or several times a week. Overall, carers of Māori gave significantly more personal care and household assistance (every day or several times a week) than did carers of non‐Māori, with non‐Māori women receiving the least personal care. Men received significantly more household assistance from their carers than did women.

### 3.3 The impact of caring

Caring impacted on the working lives of many who were employed full‐time or part‐time. Sixty‐two per cent of employed carers had made phone calls or provided care in their work time, 18 per cent had taken time in lieu or worked flexitime, and nearly one‐sixth had taken leave in order to provide care (Table 4). Forty per cent had arranged, for work reasons, another family member to provide care that they normally provided and nine per cent had paid someone else. Those in employment amounted to only half of the carers, which may account for a lack of significant differences in sex or ethnicity of care recipients.

**Table 4:** Impact of caring on informal carers by ethnic group and sex of care recipients

	Māori		Non‐Māori		*P*‐value for sex	*P*‐value for ethnicity
	Women	Men	Women	Men		
Impact on employment						
Taken annual leave, n (%)	3 (13)	3 (23)	4 (8)	8 (22)	0.06	0.5
Taken time in lieu, or worked flexitime in consultation with supervisor/colleagues, n (%)	4 (18)	5 (39)	5 (10)	8 (22)	0.05	0.1
Arranged with another family member to provide the care you normally provide, n (%)	11 (48)	7 (54)	18 (38)	12 (32)	0.7	0.271
Impact on health						
Health rated very good	29 (53)	11 (29)	41 (46)	41 (51)	0.4	0.4
EQ‐5D‐3L Utility Score^a^ mean (SD)	0.93 (0.12)	0.89 (0.14)	0.90 (0.14)	0.92 (0.13)	0.8	0.9
Overall impact on carer						
COPE^b^ positive value score, mean (SD)	14.89 (2.19)	14.32 (2.78)	15.05 (1.46)	15.04 (1.44)	0.4	0.1
COPE negative impact score, mean (SD)	8.96 (2.95)	9.70 (3.28)	8.74 (2.35)	8.90 (2.25)	0.3	0.2

Abbreviation: COPE, Carers of Older People in Europe.

a EQ‐5D‐3L is a self‐rated five dimensional health status measure, where of the maximum response represents full health and is scored as 1.0.

b COPE measures positive and negative dimensions of caregiving.

In terms of their own health, around half of the carers rated their health as very good (88%). Those caring for Māori men less often rated their health as very good, but there were no significant differences in health ratings overall in relation to the sex and ethnicity of care recipients.

There were also no significant differences in health‐related quality of life, as measured by the EQ‐5D‐3L, in relation to the sex or ethnicity of care recipients. Overall, carers were very positive about their caregiving. They scored an average of 15 out of 16 on the positive subscale of the COPE index, and only 9 out of 28 on the negative subscale with, once again, the sex or ethnicity of care recipients making no difference in terms of carers’ quality of life.

The economic value of the unpaid work done by the 261 informal carers was estimated at $4.3 million annually (Table 5). Average informal care costs were significantly higher for Māori than for non‐Māori and also higher for men than for women (estimated at $24 960 per annum for Māori men, $19 197 for Māori women, $16 382 for non‐Māori men and $10 992 for non‐Māori women).

**Table 5:** Economic value of informal caregiving for care recipients by ethnic group and sex

Informal care: all carer costs in 2015 NZD	Māori		Non‐Māori		*P*‐value for sex	*P*‐value for ethnicity
	Women	Men	Women	Men		
Average annual costs (n)	$19 197 (55)	$24 960 (38)	$10 992 (89)	$16 382 (79)	0.02	<0.001
Total annual costs	$1 055 821	$948 486	$978 277	$1 294 147		
95% confidence interval	($786 958‐$1 324 684)	($735 995‐$1 160 978)	($729 711‐$1 226 844)	($989 093‐$1 599 201)		

## 4 DISCUSSION

Our research on carers for two cohorts of Māori and non‐Māori New Zealanders in advanced age shows that sex and ethnicity are intertwined with informal caregiving in a complex manner. Sex shaped who the carers were: more often women, particularly female spouses (because of their longevity and usually younger age), and children (because sex role conventions make it more likely that women will care for parents and parents‐in‐law).^3^

Previous LiLACS NZ findings, reported elsewhere, show that sex shapes who the recipients of care are, too, with three‐quarters of men in Wave 4 receiving informal care, compared to two‐thirds of women.^5^ The previously cited NZ study also shows men receiving more care.^14^ Part of the explanation for sex differences in receipt of informal care lies in functional status of the recipients, which was not examined in this study. Previous LiLACS NZ findings showed that men in Wave 4 were less able than women to engage in activities that enable independent living, as measured by the Nottingham Extended Activities of Daily Living scale.^5^

The amount and type of care given was also shaped by sex, with men having been cared for over a longer period and receiving more hours of care overall. Patterns of sex and morbidity in later life are complex. The perceived wisdom that women have more health issues, despite greater life expectancy, is being challenged as to its applicability to older age groups.^23^ The observed pattern of men receiving more care does need further investigation, however, to establish in what ways gender may influence cultural expectations at advanced age as well as need for care. In terms of the impact of caregiving, our measures showed that neither employment status nor health of carers, nor their largely positive valuations of caring were markedly affected by the sex of those they cared for, despite the greater efforts put towards male recipients of care.^8^

Ethnicity influenced caring in very significant ways. According to our earlier study, Māori were no more likely than non‐Māori to have low functional status at Wave 4.^5^ This study showed that Māori received more hours of informal care and that their carers were younger, more likely to be offspring, more likely to live in the same household and more likely to be of different ethnicity. In relation to the latter point, it is relevant that New Zealand census data show that around half of Māori with partners have a partner who is not Māori.^24^ Both this and our earlier study, which also examined receipt of support services, suggest that Māori families provide a greater amount, a higher proportion and a higher dollar value of care than do non‐Māori families. Informal caregiving for Māori can be perceived as a cultural responsibility and is supported by wider whānau connections.^15^, ^16^ Despite giving more care, impact of care on carers was not significantly affected by the ethnicity of the care recipient.

## 5 CONCLUSION

As the balance of care continues to shift away from institutional care, it is vital to understand the social dynamics of caregiving, especially in relation to sex and ethnicity. Caregiving is a task often seen by carers in terms of being a privilege rather than a burden, but in designing support for informal carers, sex and ethnic equity should receive strong consideration.

## CONFLICT OF INTEREST

The authors declare no conflicts of interest.
